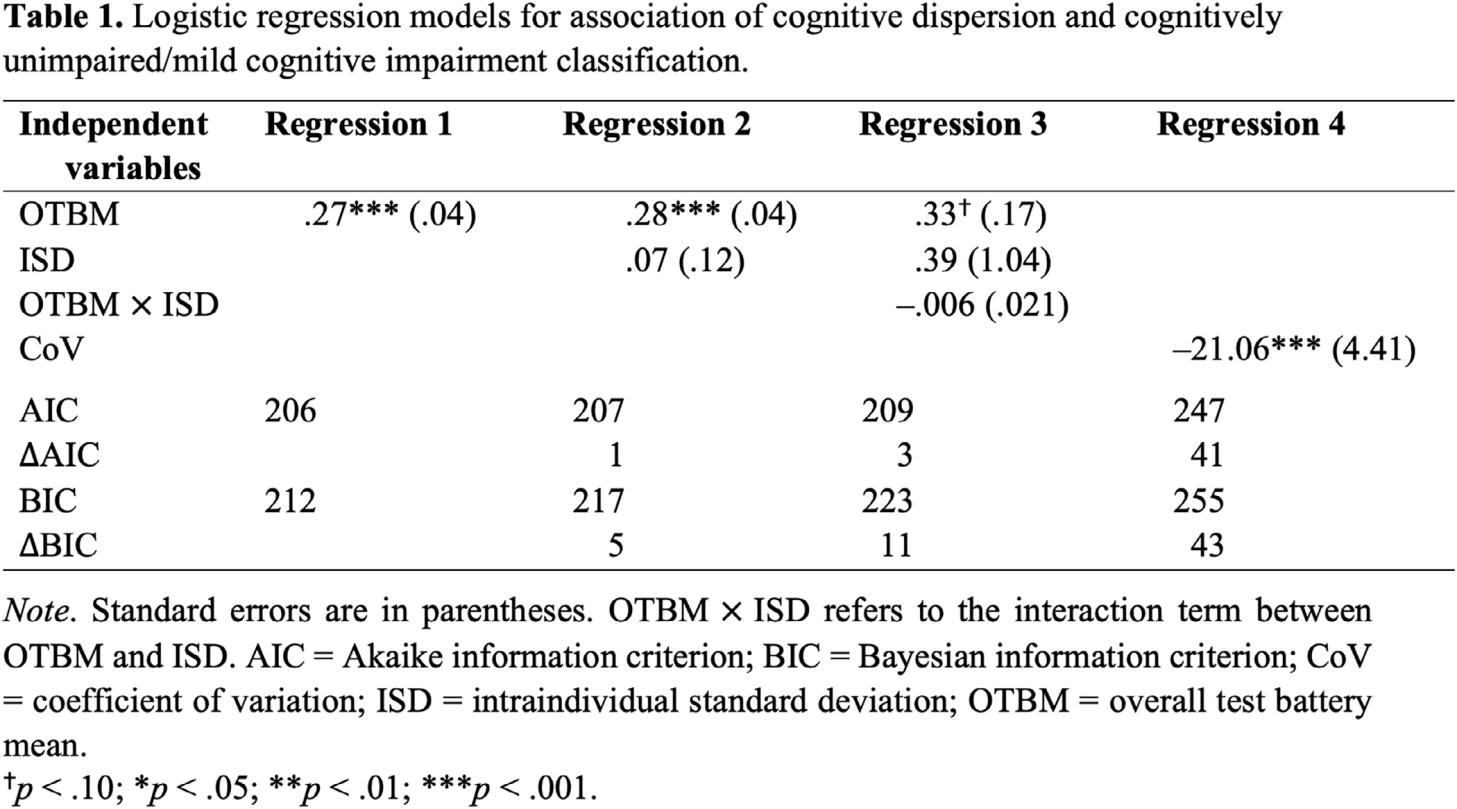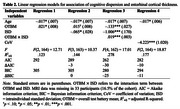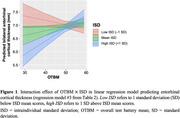# Intraindividual standard deviation vs. Coefficient of variation: a comparative analysis of late‐life cognitive dispersion

**DOI:** 10.1002/alz70857_097937

**Published:** 2025-12-24

**Authors:** Truc Tran Thanh Nguyen, Yu‐Ling Chang

**Affiliations:** ^1^ National Taiwan University, Taipei, Taiwan

## Abstract

**Background:**

Cognitive dispersion is the variability in an individual's performance across multiple neuropsychological measures. One issue that has precluded cognitive dispersion from being more widely used in clinical practice is the uncertainty over which dispersion index should be preferred, the intraindividual standard deviation (ISD) or coefficient of variation (CoV).

**Method:**

A total of 200 participants were included in this study (age 69.9 ± 6.7 years, education 13.2 ± 2.6 years, female 65%), consisting of 100 cognitively unimpaired (CU) and 100 amnestic mild cognitive impairment (MCI) participants who were age‐, sex‐, and education‐matched. For each participant, raw scores of 21 measures from 16 neuropsychological tests were converted into *T*‐scores based on the distribution of scores of the cohort. We calculated the (1) overall test battery mean (OTBM), the average of the *T*‐scores of all neuropsychological measures; (2) ISD, the SD of all *T*‐scores, with higher values reflecting higher cognitive dispersion; and (3) CoV, the ISD divided by OTBM, which is considered ISD adjusted for mean performance. Logistic and linear regression analyses were applied to examine the association between dispersion and CU/MCI classification and the bilateral entorhinal cortical thickness, respectively.

**Result:**

Using Akaike information criterion (AIC) and Bayesian information criterion (BIC) as the regression model selection criteria, we found that ISD in context of OTBM, i.e., including the interaction between OTBM and ISD as an independent variable, was more effective in predicting CU/MCI classification and entorhinal cortical thickness than CoV alone (Tables 1 and 2). Specifically, at low OTBM, participants were more likely to have greater entorhinal cortical thickness if their IIV is low, but not when their IIV is high (Figure 1). Multicollinearity was not serious in our regression models (all variance inflation factors for the effects of age, OTBM, and ISD/CoV were less than 2).

**Conclusion:**

Taken together, these results imply that ISD and CoV exhibit distinct relationships with outcomes in our study and should not be used interchangeably. Importantly, parsing out ISD and OTBM can give further insight into how these two constructs interact with one another, an information that is potentially lost when only CoV is used.